# Supplementary material for: Iterative improvement in the automatic modular design of robot swarms
Source: PeerJ Comput Sci. 2020 Dec 7;6:e322. doi: 10.7717/peerj-cs.322 (PMC7924708; doi:10.7717/peerj-cs.322)
Supplement: Supplemental Information 3 [file peerj-cs-06-322-s003.zip › argos3/doc/api/standalone/a00591_source.html]

ARGoS: plugins/simulator/entities/rotor\_equipped\_entity.h Source File


- Main Page
- Related Pages
- Namespaces
- Classes
- Files

- File List
- File Members

# plugins/simulator/entities/rotor\_equipped\_entity.h

Go to the documentation of this file.

```
00001 
00007 #ifndef ROTOR_EQUIPPED_ENTITY_H
00008 #define ROTOR_EQUIPPED_ENTITY_H
00009 
00010 #include <argos3/core/simulator/entity/entity.h>
00011 #include <argos3/core/utility/math/vector3.h>
00012 
00013 namespace argos {
00014 
00015    class CRotorEquippedEntity : public CEntity {
00016 
00017    public:
00018 
00019       ENABLE_VTABLE();
00020 
00021    public:
00022 
00023       CRotorEquippedEntity(CComposableEntity* pc_parent,
00024                            size_t un_num_rotors);
00025 
00026       CRotorEquippedEntity(CComposableEntity* pc_parent,
00027                            const std::string& str_id,
00028                            size_t un_num_rotors);
00029 
00030       virtual ~CRotorEquippedEntity();
00031 
00032       virtual void Reset();
00033 
00034       inline size_t GetNumRotors() const {
00035          return m_unNumRotors;
00036       }
00037 
00038       void SetRotor(UInt32 un_index,
00039                     const CVector3& c_position);
00040 
00041       const CVector3& GetRotorPosition(size_t un_index) const;
00042 
00043       inline const CVector3* GetRotorPositions() const {
00044          return m_pcRotorPositions;
00045       }
00046 
00047       Real GetRotorVelocity(size_t un_index) const;
00048 
00049       inline const Real* GetRotorVelocities() const {
00050          return m_pfRotorVelocities;
00051       }
00052 
00053       void SetVelocities(Real* pf_velocities);
00054 
00055       virtual std::string GetTypeDescription() const {
00056          return "rotors";
00057       }
00058 
00059    private:
00060 
00061       size_t m_unNumRotors;
00062       CVector3* m_pcRotorPositions;
00063       Real* m_pfRotorVelocities;
00064 
00065    };
00066 
00067 }
00068 
00069 #endif
```

---

Generated on 10 Jul 2018 for ARGoS by 
 1.6.1
